# Evaluation of an accelerated hydrogen peroxide disinfectant to inactivate porcine epidemic diarrhea virus in swine feces on aluminum surfaces under freezing conditions

**DOI:** 10.1186/s12917-017-1300-4

**Published:** 2017-12-01

**Authors:** Kimberlee L. Baker, Paul R. Thomas, Locke A. Karriker, Alejandro Ramirez, Jianqiang Zhang, Chong Wang, Derald J. Holtkamp

**Affiliations:** 1 Department of Veterinary Diagnostic and Production Animal Medicine, Iowa State University College of Veterinary Medicine, 1809 S Riverside Dr, Ames, IA USA; 2 AMVC Management Services, 1786 190th St, Audubon, IA USA; 3 Swine Medicine Education Center, Iowa State University College of Veterinary Medicine, 1809 S Riverside Dr, Ames, IA USA

**Keywords:** Swine, PEDV, Disinfection, Temperature, Accelerated hydrogen peroxide

## Abstract

**Background:**

Since its emergence in 2013, porcine epidemic diarrhea virus (PEDV) spread rapidly throughout the country due, in part, to contaminated livestock trailers. The objective of this study was to test the efficacy of an accelerated hydrogen peroxide (AHP) disinfectant for inactivating PEDV in swine feces on metal surfaces under freezing conditions. One 15.24 X 15.24 X 2.54 cm aluminum coupon, contaminated with swine feces, and randomly matched to one pig was the experimental unit. Eight treatment groups representing two AHP concentrations (1:16 and 1:32) in a 10% propylene glycol solution, two contact times in a -10 °C freezer (40 min and 60 min), and two levels of fecal contamination (5 mL and 10 mL) in addition to negative and positive control groups were evaluated. Forty 3-week-old pigs, intragastrically inoculated with the contents of the coupons after treatment, were used as a bioassay to determine the infectivity of PEDV after treatment. Infectivity was determined by detection of virus with a nucleocapsid (N) gene-based quantitative real-time reverse transcription polymerase chain reaction (RT-qPCR) on rectal swabs collected from the inoculated pigs on days three and seven post-inoculation.

**Results:**

All post-treatment swabs from the negative control coupons were negative for PEDV via RT-qPCR. All post-treatment swabs collected from coupons in the AHP disinfectant treatment groups and the positive control group were positive for PEDV via RT-qPCR. For the bioassay, no rectal swabs from pigs in the negative control (0 of 4) or the AHP disinfectant treatment groups (0 of 32) were positive for PEDV. Rectal swabs from all pigs within the positive control group (4 of 4) were positive for PEDV by RT-qPCR.

**Conclusions:**

Under the conditions of this study, 1:16 and 1:32 dilutions of the AHP disinfectant successfully inactivated PEDV in swine feces on metal surfaces when applied at -10 °C with 40 or 60 min of contact time. This study also suggests that a positive RT-qPCR result for PEDV on an environmental sample should be expected when the AHP disinfectant is applied under freezing conditions, but does not necessarily indicate that an infectious dose of PEDV remains after disinfection.

**Electronic supplementary material:**

The online version of this article (doi: 10.1186/s12917-017-1300-4) contains supplementary material, which is available to authorized users.

## Background

Porcine epidemic diarrhea (PED) was first described in 1971 as cases of profuse, watery diarrhea affecting all ages of pigs in England [1]. In 1978, a novel coronavirus, porcine epidemic diarrhea virus (PEDV), was determined to be the causative agent [2, 3]. The first cases of PEDV in the United States were confirmed during May 2013 from farms in Iowa and Indiana [4]. PEDV spread rapidly throughout the United States after its introduction mainly due to the ease of transmission via fecal-oral and pig-to-pig contact [5]. Livestock trailers that haul pigs to and from collection points such as livestock auctions or harvest facilities have been implicated as mechanical vectors for PEDV [6]. Contaminated livestock trailers likely pose a significant risk for PEDV transmission and movement throughout the country.

This risk has historically been mitigated through trailer sanitation and decontamination procedures developed for porcine reproductive and respiratory syndrome virus (PRRSV) when high pressure washing alone was found insufficient to inactivate PRRSV [7–10]. Because disinfection and drying are more effective when applied to a clean trailer with little or no remaining organic matter, the industry standard for sanitation and decontamination of livestock trailers includes trailer washing, disinfection, and drying, either naturally or with a thermo-assisted drying and decontamination (TADD) system [7–9]. Similar research on PEDV demonstrated that the industry standard wash, disinfection, and dry successfully inactivated PEDV on metal surfaces when detergent and a combination of quaternary ammonium and glutaraldehyde disinfect were used [11].

The industry standard wash, disinfect, and dry is always the best method for livestock trailer sanitation and decontamination. For this reason, swine producers should always strive to use the industry standard sanitation and decontamination protocols on all livestock trailers between loads. However, a complete wash, disinfect, and dry requires an investment in time, logistics, and specialized facilities which deters some swine producers and contract haulers from performing the industry standard protocols between every load of pigs hauled. Consequently, a significant number of pigs, especially market pigs, are hauled on trailers that are not subjected to any sanitation or decontamination procedures between loads, posing a significant risk. Successfully decreasing the risk of PEDV transmission from contaminated livestock trailers may depend on the development of cost-effective sanitation procedures, as an alternative to doing nothing, which can be completed in a short period of time without specialized facilities. In addition, identification of disinfectants and other decontamination processes that work in the presence of some organic matter may decrease the risk of viral transmission from livestock trailers that are washed, but sanitation and decontamination procedures are not closely monitored or performed poorly.

Recent PEDV research demonstrated that holding a metal surface contaminated with PEDV positive feces at 71 °C for 10 min or 20 °C for 7 days was efficacious at inactivating PEDV [12]. An accelerated hydrogen peroxide® (AHP®) disinfectant (Intervention®, Virox Technologies Inc., Oakville, Ontario, Canada) successfully inactivated PEDV in the presence of fecal contamination on metal surfaces with a 30-min contact time at 20 °C. The concentrated form of the AHP disinfectant was efficacious at dilutions of 1:16 and 1:32 in the presence of feces [13]. Intervention® is labeled as virucidal at dilution rates of 1:16 to 1:64 in the presence of 200 ppm hard water and 5% serum load with a 5-min contact time. It contains anionic surfactants, nonionic surfactants, and stabilizers that help improve the stability and microbicidal action of hydrogen peroxide [14, 15]. PEDV outbreaks, however, tend to be more prevalent in the cooler winter months where a complete wash, disinfect, and dry is more difficult to complete due to freezing temperatures. Water along with most aqueous disinfectants freeze around 0 °C; making trailer sanitation and decontamination difficult. It has been previously demonstrated that diluting a quaternary ammonium and glutaraldehyde combination disinfectant (Synergize; Preserve International, Atlanta, Georgia) in either a 10% propylene glycol (PG) or 40% methanol solution prevented freezing and allowed the disinfectant to inactivate PRRSV at temperatures below 0 °C [10].

The objective of this study was to evaluate two concentrations of an AHP disinfectant in a 10% PG solution to determine if the mixture was sufficient to inactivate PEDV in the presence of swine feces on metal surfaces at -10 °C. Conditions were chosen to mimic those found in commercial livestock trailers in winter months after most of the fecal and organic matter has been removed by scraping and a traditional wash is unavailable.

## Methods

### Experimental design

The experimental unit was a single aluminum coupon contaminated with swine feces matched to an individual 3-week old pig. The pig was intragastrically inoculated with the contents of the coupon post treatment, as a bioassay to determine if the treatment applied to the contaminated coupon effectively inactivated PEDV. Three-week old pigs were used in this study because they are relatively susceptible to infection with PEDV, but mortality is rare. Previous work reported that 100% of 21-day-old pigs inoculated with 10 mL of a virulent PEDV prototype isolate with titers of 5.6–560 TCID_50_/ml were infected, but pigs inoculated with lower titers (0.0056–0.56 TCID_50_/ml) of PEDV were not infected [16].

The primary outcome variable was the proportion of pigs in each treatment group that were PEDV-positive by bioassay to determine if infectious PEDV was present after the disinfectant treatment. The null hypothesis was that there was no difference between the positive control group and the disinfectant treatment groups in the proportion of pigs infected with PEDV after being inoculated with the material collected from the coupons. The bioassay result was determined by nucleocapsid (N) gene-based quantitative real-time reverse transcription polymerase chain reaction (RT-qPCR) performed at the Iowa State University Veterinary Diagnostic Laboratory (ISU VDL) on rectal swabs collected from each pig on days three and seven post-inoculation. The primers and probe of the PEDV RT-qPCR were previously described [6, 16, 17]. Each PCR was set up and performed in accordance with previously described procedures [13, 16, 17]. Current viral culture methods make it difficult to culture wild-type PEDV outside of an animal model. Therefore, to determine if live infectious PEDV is present in a sample, a bioassay using an animal model remained the best alternative. The use of a bioassay also eliminated questions about the cytotoxic impact feces and disinfectant may have had on the outcome of virus isolation.

Personnel performing disinfectant treatments, necropsies, and collecting samples were not blinded to the treatment groups. Blinding these individuals was not possible because all procedures were performed in a specific order, starting with the negative control group and ending with the positive control group, to minimize the risk of transmitting PEDV between treatment groups. Laboratory personnel that performed the RT-qPCR testing for PEDV and personnel performing the statistical analyses were blinded to the treatment groups.

### Coupons

Forty 15.24 cm X 15.24 cm X 2.54 cm aluminum coupons were manufactured using aluminum with a material thickness of 0.32 cm, resembling the type of material found in livestock trailers. These coupons were used in previous studies evaluating the efficacy of other sanitation and decontamination procedures for PEDV inactivation [11–13]. To simulate the cleaning action of the AHP disinfectant and runoff seen in commercial livestock trailers as the AHP disinfectant is transformed from foam to a liquid, six 8 mm diameter holes were drilled at the junction of the bottom and sidewall of the coupon.

### Treatment groups

Two volumes of fecal contamination (5 mL and 10 mL); two concentrations of AHP disinfectant (1:16 and 1:32) prepared in a solution that was 10% PG by volume; and two contact times (40 min and 60 min) were evaluated. A positive control and negative control group were also included (Table 1). Forty minutes of contact time was chosen by applying the Arrhenius equation which in previous work led to the conclusion that for every 10 °C decrease in temperature the contact time of a disinfectant doubles [18]. PEDV-positive feces were used to contaminate coupons in the positive control group (B) and all treatment groups (C through J). PEDV negative feces were used to contaminate coupons in the negative control group (A). The negative (A) and positive (B) control groups were not sham disinfected. The AHP disinfectant used in this study was applied as a thick foam which persisted for the duration of the contact time and had minimal rinsing and diluting effects. The best candidate for a sham disinfectant would be a non-disinfecting solution that produced a persistent foam similar to that of the AHP disinfectant; however, after extensive research and pre-trial work, a suitable non-disinfecting foam was not identified by the investigators. Using a non-foam liquid for sham disinfection would result in a greater rinsing and diluting effect as the liquid would run out of the holes in the coupons at a faster rate than the persistent foam produced by the AHP disinfectant would; therefore, sham disinfection was not done. Four replicates of aluminum coupons were included for each treatment group.

**Table 1:** Description of treatment groups (4 replicates per treatment group)

Treatment group	Volume and PEDV status of feces	Disinfectant and concentration	Contact time at -10 °C
(A) Negative Control	5 mL PEDV-negative feces	None	None
(B) Positive Control	5 mL PEDV-positive feces	None	40 min
(C) Light, 1:32, 40 min	5 mL PEDV-positive feces	AHP at 1:32	40 min
(D) Heavy, 1:32, 40 min	10 mL PEDV-positive feces	AHP at 1:32	40 min
(E) Light, 1:16, 40 min	5 mL PEDV-positive feces	AHP at 1:16	40 min
(F) Heavy, 1:16, 40 min	10 mL PEDV-positive feces	AHP at 1:16	40 min
(G) Light, 1:32, 60 min	5 mL PEDV-positive feces	AHP at 1:32	60 min
(H) Heavy, 1:32, 60 min	10 mL PEDV-positive feces	AHP at 1:32	60 min
(I) Light, 1:16, 60 min	5 mL PEDV-positive feces	AHP at 1:16	60 min
(J) Heavy, 1:16, 60 min	10 mL PEDV-positive feces	AHP at 1:16	60 min

### Contamination and disinfection procedures

The feces used to contaminate the coupons were obtained from a previous experiment where 3-week-old pigs were inoculated with PEDV isolate US/Iowa/18984/2013 [17]. Feces were collected from pigs, confirmed to be positive for PEDV by RT-qPCR, 7 days post inoculation, which was within the peak viral shedding timeframe [17]. After collection, feces from individual pigs were stored at -80 °C. On study day 0, the feces from individual pigs were thawed and pooled into a single fecal homogenate to ensure that the amount of PEDV and composition of the feces was uniform for each replicate. Samples from each replicate were tested at the ISU VDL by RT-qPCR. The quantitative genomic copies/mL ranged from 10^8.00^ to 10^9.06^ genomic copies/mL across all replicates (Table 2 and Additional file 1). PEDV negative feces were obtained from the negative control pigs in a previous study [13]. Fecal collection and storage procedures were the same as those for the PEDV positive feces. Prior to freezing, a sample of the PEDV negative feces was submitted to the ISU VDL to confirm its PEDV negative status. Diagnostic testing confirmed that the sample was negative for PEDV by RT-qPCR.

**Table 2:** Summary of PEDV RT-qPCR results for the pre-treatment and post-treatment swabs

Treatment group	Pre-treatment Ct Value and (genomic copies/mL)	Percentage positive or suspect for PEDV	Post-treatment Ct Value and (genomic copies/mL)	Percentage positive or suspect for PEDV
(A) Neg Control	>35 (0) >35 (0) >35 (0) >35 (0)	0% (0 of 4)	>35 (0) >35 (0) >35 (0) >35 (0)	0% (0 of 4)
(B) Pos Control	18.2 (10^8.56^) 19.0 (10^8.32^) 19.5 (10^8.17^) 19.2 (10^8.26^)	100% (4 of 4)	18.6 (10^8.44^) 17.1 (10^8.88^) 18.8 (10^8.38^) 18.8 (10^8.38^)	100% (4 of 4)
(C) Light, 1:32, 40 mins	17.2 (10^8.85^) 17.4 (10^8.79^) 17.9 (10^8.64^) 17.6 (10^8.73^)	100% (4 of 4)	27.1 (10^5.94^) 21.0 (10^7.73^) 29.1 (10^5.35^) 28.6 (10^5.49^)	100% (4 of 4)
(D) Heavy, 1:32, 40 mins	16.8 (10^8.97^) 18.0 (10^8.61^) 17.8 (10^8.67^) 18.1 (10^8.58^)	100% (4 of 4)	24.6 (10^6.67^) 19.2 (10^8.26^) 24.0 (10^6.85^) 20.6 (10^7.85^)	100% (4 of 4)
(E) Light, 1:16, 40 mins	18.7 (10^8.41^) 17.9 (10^8.64^) 18.2 (10^8.56^) 18.7 (10^8.41^)	100% (4 of 4)	33.6 (10^4.02^) 31.1 (10^4.76^) 28.9 (10^5.41^) 30.7 (10^4.88^)	100% (4 of 4)
(F) Heavy, 1:16, 40 mins	17.2 (10^8.85^) 17.8 (10^8.67^) 18.5 (10^8.47^) 18.2 (10^8.56^)	100% (4 of 4)	26.1 (10^6.23^) 23.0 (10^7.14^) 23.9 (10^6.88^) 23.0 (10^7.14^)	100% (4 of 4)
(G) Light, 1:32, 60 mins’	18.0 (10^8.61^) 17.6 (10^8.73^) 17.0 (10^8.91^) 18.2 (10^8.56^)	100% (4 of 4)	27.5 (10^5.82^) 26.1 (10^6.23^) 23.6 (10^6.97^) 27.3 (10^5.88^)	100% (4 of 4)
(H) Heavy, 1:32, 60 mins	17.6 (10^8.73^) 17.9 (10^8.64^) 18.7 (10^8.41^) 17.0 (10^8.91^)	100% (4 of 4)	26.1 (10^6.23^) 23.7 (10^6.94^) 21.5 (10^7.59^) 24.8 (10^6.61^)	100% (4 of 4)
(I) Light, 1:16, 60 mins	17.6 (10^8.73^) 17.5 (10^8.76^) 17.8 (10^8.67^) 17.9 (10^8.64^)	100% (4 of 4)	34.5 (10^3.76^) 33.4 (10^4.08^) 34.7 (10^3.70^) 32.3 (10^4.40^)	100% (4 of 4)
(J) Heavy, 1:16, 60 mins	16.5 (10^9.06^) 18.0 (10^8.61^) 17.3 (10^8.82^) 20.1 (10^8.00^)	100% (4 of 4)	28.0 (10^5.67^) 24.9 (10^6.58^) 24.7 (10^6.64^) 24.9 (10^6.58^)	100% (4 of 4)

**Fig. 1:**
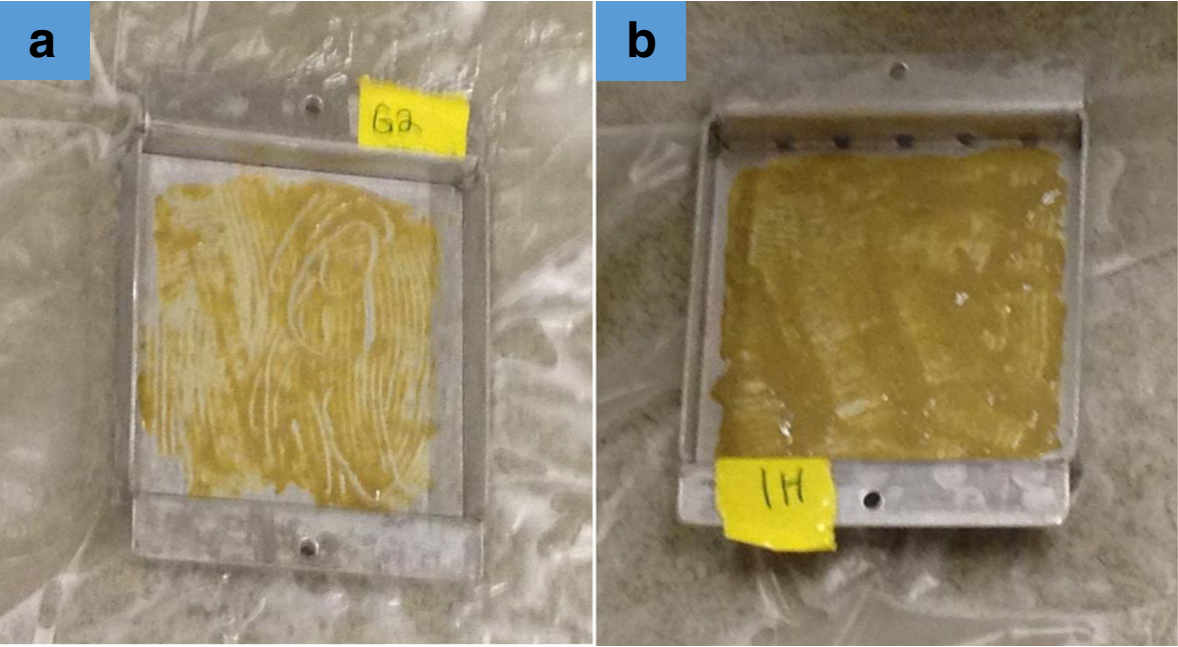
Fecal contamination of aluminum coupons. Feces were applied in an even layer to aluminum coupons using an adhesive spreader. Five mL of feces were applied to coupons in treatment groups **A**, **B**, **C**, **E**, **G**, and **I**. Ten mL of feces were applied to coupons in treatment groups **D**, **F**, **H**, and **J**. One representative coupon in group G (**a**) and group H (**b**) is shown

The in-vivo portion of the study was initiated on study day 0. Prior to contamination and treatment of the coupons, 2 mm thick plastic sheeting was placed on the floor. The plastic sheeting was changed and the floor under the plastic sheeting was disinfected with Virkon™ S disinfectant (Lanxess, Wilmington, DE, USA) between each treatment group to reduce the risk of cross contamination. For the negative control group (A), 5 mL of PEDV negative feces were applied to four aluminum coupons. Five mL of PEDV positive feces was applied to all coupons in groups B, C, E, G, and I. Ten mL of PEDV positive feces were applied to all coupons in groups D, F, H, and J (Fig. 1). For all study groups (A through J), contamination of the coupons with feces was performed using a disposable hard plastic spreader sold in hardware stores to spread adhesive on floors. A new adhesive spreader was used on each coupon to prevent cross-contamination between replicates. Five mL and 10 mL of feces, when spread evenly over the floor of each coupon, resulted in an even layer that was ≤2 mm and were chosen to reflect the range of organic matter remaining in the interior of a commercial livestock trailer after it has been manually scraped to remove bedding and feces. Following contamination with feces, all coupons were individually sampled using a commercial swab and transport system. The pre-treatment swabs were submitted to the ISU VDL to test for the presence of PEDV by RT-qPCR.

Following fecal contamination, coupons, except those assigned to the negative control group A, were pre-cooled in a commercial refrigerator, set to 4 °C for 30 min. This pre-cooling period was designed to mimic conditions in a scraped livestock trailer after transporting pigs during the winter months. Plastic sheeting was placed on the refrigerator’s shelves and changed between each treatment group to prevent cross-contamination.

After the pre-cooling period, AHP disinfectant solution was applied to the contaminated coupons in the treatment groups C through J. The AHP disinfectant was prepared by diluting a 4.25% concentrate of the AHP disinfectant (Intervention®, Virox Technologies Inc., Oakville, Ontario, Canada) with tap water from a municipal water source and PG. The final AHP disinfectant solution contained 10% PG and a ratio of AHP concentrate to final solution of 1:16 for treatment groups E, F, I, and J and 1:32 for treatment groups C, D, G, and H. PG is an organic solvent that can be used as a safe anti-freezing agent when mixed with phenol, quaternary ammonium, and quaternary ammonium formaldehyde disinfectants without reducing their efficacy [19]. Coupons in the negative control (A) and positive control (B) groups were not sham disinfected. A liquid volume of approximately 30 mL of AHP disinfectant solution was applied as a foam to all 4 coupons in each treatment group (C through J) using a 5.7 L pump-up foamer (model #A8020A, Ogena Solutions, LLC, Stoney Creek, ON, Canada). To disinfect a 15.8 m double-decked livestock trailers, 189 L of AHP disinfectant would be applied over a 10-min period using a proportioning foamer with a flow rate of 18.9 L per minute. Based on the area of the coupons used in this study, 30 mL of AHP disinfectant was determined proportionally equivalent to the 189 L used on livestock trailers. Using the same 5.7 L foamer, a series of timed applications were performed prior to study initiation to establish that a 3 s application time was required to apply the 30 mL of AHP disinfectant solution.

Following treatment with AHP disinfectant solution, the coupons were placed in a freezer set at −10 °C for their allotted contact time as described in Table 1. To prevent cross contamination between treatments, the freezer drawers were lined with 2 mm plastic sheeting and a folded bath towel was placed on top of the plastic to absorb any liquid runoff from the coupons. To prevent replicates within the treatment group from cross-contaminating each other, each coupon was placed into a 16 cm X 16 cm X 3 cm pan crafted from aluminum foil. New plastic, towels and aluminum foil pans were used for every treatment group. Coupons in the positive control group (B) were also placed in the freezer for 40 min to confirm that the time at -10 °C alone was not responsible for PEDV inactivation. Coupons in the negative control group (A) were not placed in the freezer.

Ten minutes after contamination of the coupons in the negative control group (A), a post-treatment swab was taken using a commercial swab and transport system. For all other study groups (B through J), the swabs were collected following the treatment described in Table 1. All swabs collected post-treatment were submitted to the ISU VDL and tested for the presence of PEDV RNA by RT-qPCR.

**Fig. 2:**
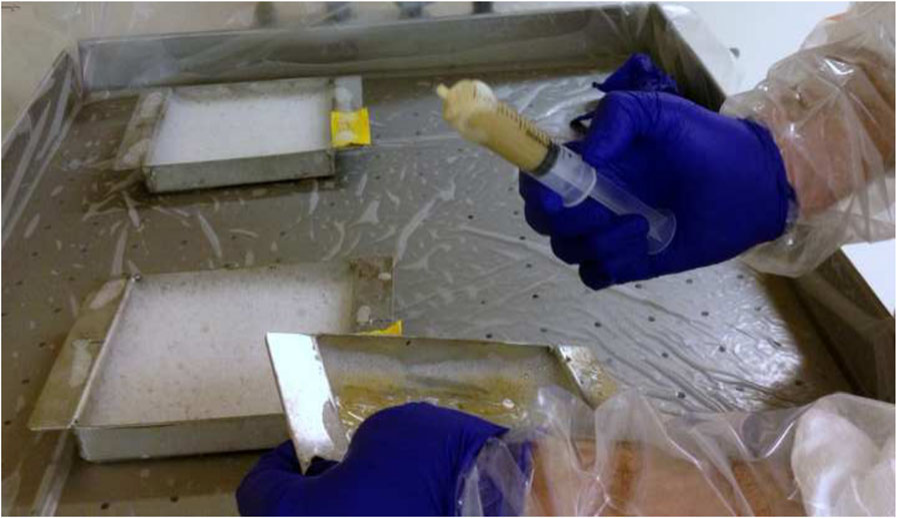
Collection of feces/AHP disinfectant homogenate used to inoculate a pig for the bioassay. A mixture of feces, AHP disinfectant, PG, and saline was recollected from the coupon using a 20 mL syringe. This syringe was labeled with the coupon identification number and matched to a single pig for the bioassay

After the post-treatment swab was collected, the coupon was tipped away from the holes and 10 mL of sterile 0.9% sodium chloride (saline) solution was added using a new 12 mL syringe for each coupon. A coupon dedicated toothbrush was used to re-suspend the feces/AHP disinfectant/PG/saline mixture; creating a homogenate sample suitable for recollection as inoculum. The resultant homogenate was collected using a 20-mL syringe (Fig. 2). During recollection of the inoculum sample, nitrile gloves were changed between each coupon to prevent cross contamination between replicates and 2 mm plastic sheeting was placed under the coupons and changed between treatment groups to prevent cross-contamination between treatments.

### Swine bioassay

#### Source of animals and housing

The experimental protocol was approved by the Iowa State University Institutional Animal Care and Use Committee (Log Number: 11–14-7904-S) and the Iowa State University Institutional Biosafety Committee (Log Number 14-I-0040-A) prior to initiation of any experimental activity. Forty 3-week-old commercial pigs, 17 barrows and 23 gilts, were obtained from WeSearch, LLC in Iowa and housed in the Iowa State University Veterinary Medical Research Institute for the duration of the study. Upon delivery (study day −4), all pigs (40/40) were individually identified with a unique ID number on a plastic ear tag and weighed. Pigs were blocked by weight and then randomly assigned within each weight group to one of the 10 treatment groups (Table 1) using the RAND function in Microsoft Excel (version 2010).

On study day −1, serum and rectal swabs were collected from the pigs to confirm their PEDV negative status. Eight mL of whole blood was collected from each pig via jugular venipuncture and transferred to an 8.5 mL serum separator tube and centrifuged at 3100 g for 8 min. Serum was submitted to the ISU VDL for diagnostic testing. Serum samples were pooled (5 samples per pool) and tested for porcine reproductive and respiratory virus (PRRSV) via RT-qPCR. Individual serum samples were tested for antibodies to PRRSV using a commercial PRRS X3 Ab Test (IDEXX Laboratories, Inc., Westbrook, Maine), and for antibodies to transmissible gastroenteritis virus (TGEV) and porcine respiratory corona virus (PRCV) by a commercial TGEV/PRCV-Ab differential enzyme-linked immunosorbent assay (ELISA, SVANOVA Biotech AB, Uppsala, Sweden). Individual serum samples were also screened for antibodies to PEDV by indirect fluorescent antibody (IFA) assay following previously described procedures [16]. Rectal swabs were obtained via the same commercial swab and transport system used to swab the coupons. Individual rectal swabs were tested for TGEV, porcine rotaviruses (groups A, B and C), and PEDV by real time RT-PCRs at the ISU VDL.

Each treatment group was designated one room. The four pigs in each group were housed individually in a raised tub described in detail in previous studies [12, 13]. Individual housing was accomplished using solid transparent dividers, creating four compartments within one tub. The transparent dividers allowed visual and auditory contact between the pigs, but prevented nose-to-nose and fecal-oral contact. Urine and feces fell through a grate at the bottom of the tub and drained into a bucket to minimize the risk of contaminating the room’s floor. Each section of the tub had designated water and feed sources. During the study, each pig was assigned its own bag of feed and feed cup to minimize cross contamination. Previous studies demonstrated that this housing method successfully prevented transmission of PEDV between pigs in a group [12]. Pigs were fed an age appropriate, pelleted, starter diet ad libitum. The diet consisted primarily of corn and soybean meal and did not contain porcine derived products.

#### Inoculation of pigs with contents of coupon post-treatment

On study day 0, the homogenate of feces, AHP disinfectant, PG and saline, collected from each coupon as described above, was immediately used to inoculate pigs for the bioassay via oral gastric gavage with a 14 French rubber catheter as previously described [12]. Each pig was inoculated with the contents of its designated coupon, with PEDV RT-qPCR results ranging from 10^3.70–^10^8.88^ genomic copies/mL for coupons assigned to treatment groups B – J (Table 2 and Additional file 1). To prevent cross contamination between treatment groups and replicates within the same treatment, study personnel followed a strict biosecurity protocol used successfully in previous work [12]. Disposable Tyvek coveralls (DuPont, USA) and respirators were worn by personnel at all times and were changed between each treatment group. Coveralls were inspected after each inoculation and were changed between pigs if inoculum or fecal material was present. Furthermore, nitrile gloves and arm-length disposable obstetrical sleeves were worn and changed between each pig to prevent cross-contamination between replicates.

Pigs were monitored daily, on study days 0 through 7, for clinical signs consistent with PEDV by the same study investigator. On days 3 and 7 post-inoculation, rectal swabs were collected from each pig using a commercial swab and transport system. To avoid cross contamination between replicates, pigs were not removed from their compartment and personnel used the same biosecurity procedures as previously described for inoculation. A bioassay was considered positive if the rectal swab was PEDV-positive by RT-qPCR (Ct value less than 35) on study day 3 or study day 7. A bioassay was considered negative if both rectal swabs (study days 3 and 7) were PEDV-negative by RT-qPCR (Ct > 35).

On study day 7, all pigs were humanely euthanized using a penetrating captive bolt gun and necropsied. During necropsy, all organ systems were evaluated and any gross lesions or abnormal pathology was noted. Fresh and 10% formalin-fixed samples of mesenteric lymph nodes, ileum, and jejunum and fresh cecal and spiral colon contents were collected from each pig. All fresh samples were placed in a -80 °C freezer and all samples were held in the event further testing might be required to confirm the results of the swine bioassay.

All statistical analyses were performed using SAS® (Enterprise Guide 5.1; SAS Institute, Cary, NC, USA). A Fisher’s Exact Test was used to evaluate pairwise differences in the proportion of pigs positive by bioassay between all 10 treatment groups. Ct values were analyzed using two way analyses of variance (ANOVA) models with treatment, time (pre versus post) and their interaction. Pre-treatment Ct values were compared between groups using an F-test. Differences in Ct values between pre- and post-treatment were assessed for each study group using a two-sided T-Test. A *p* value <0.05 was considered statistically significant.

## Results

### Pre-trial diagnostic screening

Serum and fecal samples obtained on study day −1 confirmed that all 40 pigs were negative for PEDV by RT-qPCR as well as negative for antibodies to PEDV via IFA. Additionally, all pigs were negative for PRRSV, porcine rotaviruses (groups A, B, and C), and TGEV by RT-qPCR and for antibodies to TGEV via differential ELISA. Fifteen pigs were positive, 23 were suspect, and 2 were negative for antibodies to PRCV by differential ELISA. Three pigs were positive for antibodies to PRRSV via ELISA.

### Coupon swabs

PEDV RT-qPCR results from swabs taken from fecal contaminated aluminum coupons before and after treatment with AHP disinfectant are displayed in Table 2 and Additional file 1. In the negative control (A), all pre-treatment swabs, taken immediately after PEDV negative feces were applied to the four coupons, were negative for PEDV RNA by RT-qPCR. All pre-treatment swabs, taken immediately after PEDV positive feces were applied to coupons, from the positive control (B) were positive for PEDV RNA by RT-qPCR with quantitative results ranging from 10^8.17^–10^8.56^ genomic copies/mL. Likewise, all swabs obtained after fecal contamination but prior to treatment from the coupons in groups C – J were PEDV-positive via RT-qPCR with quantitative results from 10^8.00^–10^9.06^ genomic copies/mL. There were no significant differences in pre-treatment Ct values from the coupons assigned to the eight disinfectant (C – J) and the positive control (B) groups (*p*-value = 0.9435).

Post-treatment swabs (4 of 4) from the negative control (A) coupons, taken 10 min after PEDV negative feces were applied to the coupon, were negative for PEDV RNA by RT-qPCR. All (4 of 4) post-treatment swabs from the positive control (B) coupons, taken after pre-cooling and 40 min in a -10 °C freezer, were positive for PEDV RNA via RT-qPCR with quantitative results ranging from 10^8.38^–10^8.88^ genomic copies/mL. All (32 of 32) post-treatment swabs collected from coupons in the AHP disinfectant treatment groups C – J were positive for PEDV RNA via RT-qPCR with quantitative results ranging from 10^3.76^–10^8.26^ genomic copies/mL. The difference in Ct values between pre- and post-treatment were significantly different than zero (*p* value <0.0001) for all of the AHP disinfectant treatment groups (C – J).

### Swine bioassay

PEDV RT-qPCR results for rectal swabs taken 3 and 7 days post inoculation and the final swine bioassay results by treatment group are displayed in Table 3 and Additional file 1. Rectal swabs collected from all pigs in the negative control (A) and AHP disinfectant treatment groups C – J on study days 3 and 7 were negative for PEDV by RT-qPCR. Rectal swabs from all four pigs in the positive control (B) collected on study days 3 and 7 were positive for PEDV by RT-qPCR. The proportion of pigs positive by bioassay for the negative control (A) and all of the AHP disinfectant treatment groups (C – J) were significantly different than proportion of pigs positive in the positive control (B) group via Fishers Exact Test (*p*-value <0.05).

**Table 3:** Post-inoculation rectal swab PEDV N-gene RT-qPCR and swine bioassay results

Treatment Group	Day 3 Rectal Swab; Individual CT results (genomic copies / mL)	Day 3 Rectal Swab; Percentage positive for PEDV RNA	Day 7 Rectal Swab; Individual CT Results (genomic copies / mL)	Day 7 Rectal Swab; Percentage positive for PEDV RNA	Swine Bioassay Result; Percentage positive for PEDV RNA
(A) Negative Control	>35 (0) >35 (0) >35 (0) >35 (0)	0% (0 of 4)	>35 (0) >35 (0) >35 (0) >35 (0)	0% (0 of 4)	0% (0 of 4)^a^
(B) Positive Control	16.2 (10^9.14)^ 15.1 (10^9.46^) 16.8 (10^8.96^) 15.0 (10^9.49^)	100% (4 of 4)	22.2 (10^7.37^) 15.2 (10^9.43^) 19.3 (10^8.23^) 17.4 (10^8.79^)	100% (4 of 4)	100% (4 of 4)^b^
(C) Light, 1:32, 40 mins	>35 (0) >35 (0) >35 (0) >35 (0)	0% (0 of 4)	>35 (0) >35 (0) >35 (0) >35 (0)	0% (0 of 4)	0% (0 of 4)^a^
(D) Heavy, 1:32, 40 mins	>35 (0) >35 (0) >35 (0) >35 (0)	0% (0 of 4)	>35 (0) >35 (0) >35 (0) >35 (0)	0% (0 of 4)	0% (0 of 4)^a^
(E) Light, 1:16, 40 mins	>35 (0) >35 (0) >35 (0) >35 (0)	0% (0 of 4)	>35 (0) >35 (0) >35 (0) >35 (0)	0% (0 of 4)	0% (0 of 4)^a^
(F) Heavy, 1:16, 40 mins	>35 (0) >35 (0) >35 (0) >35 (0)	0% (0 of 4)	>35 (0) >35 (0) >35 (0) >35 (0)	0% (0 of 4)	0% (0 of 4)^a^
(G) Light, 1:32, 60 mins	>35 (0) >35 (0) >35 (0) >35 (0)	0% (0 of 4)	>35 (0) >35 (0) >35 (0) >35 (0)	0% (0 of 4)	0% (0 of 4)^a^
(H) Heavy, 1:32, 60 mins	>35 (0) >35 (0) >35 (0) >35 (0)	0% (0 of 4)	>35 (0) >35 (0) >35 (0) >35 (0)	0% (0 of 4)	0% (0 of 4)^a^
(I) Light, 1:16, 60 mins	>35 (0) >35 (0) >35 (0) >35 (0)	0% (0 of 4)	>35 (0) >35 (0) >35 (0) >35 (0)	0% (0 of 4)	0% (0 of 4)^a^
(J) Heavy, 1:16, 60 mins	>35 (0) >35 (0) >35 (0) >35 (0)	0% (0 of 4)	>35 (0) >35 (0) >35 (0) >35 (0)	0% (0 of 4)	0% (0 of 4)^a^

^a,b^ Different superscripts indicate statistically significant differences (*p*-value <0.05) by a Fishers Exact Test

## Discussion

Under freezing conditions (−10 °C) an AHP disinfectant prepared in a 10% PG solution inactivated PEDV in the presence of feces on metal surfaces. Both AHP disinfectant dilutions (1:16 and 1:32), and contact times (40 min and 60 min) evaluated were sufficient to inactivate PEDV at either fecal load (5 mL and 10 mL), under the conditions of this study. Bioassay results from this study support previous work where the same AHP disinfectant successfully inactivated PEDV in swine feces on metal surfaces at 20 °C [13]. Findings from this study demonstrate that manually removing the organic material from a trailer via scraping and then applying an AHP disinfectant may be an effective alternative to doing nothing between loads, year round. Additionally, mixing the AHP disinfectant in a 10% PG solution prevented the disinfectant solution from freezing during 40 or 60 min of contact time in a freezer set to -10 °C, supporting previously published data on utilizing PG as an anti-freezing agent [10, 19].

In contrast to previous work [13], all post-treatment swabs (32 of 32), from the coupons contaminated with PEDV positive feces and then subjected to one of eight AHP disinfectant treatments (groups C-J) at -10 °C, were positive for the presence of PEDV RNA by RT-qPCR. However, none of the coupons (0 of 32) contained infectious virus as demonstrated by swine bioassay. While swine veterinarians sometimes use RT-PCR to evaluate the success of sanitation and decontamination protocols in practice, its major limitation is that it does not differentiate between infectious virus and noninfectious fragments of PEDV RNA. These results indicate that the AHP disinfectant inactivated the virus while leaving a sufficient amount of genetic material intact to interact with the primers used in the RT-qPCR assay. Hydrogen peroxide is an oxidizing agent that inactivates viruses by denaturing viral proteins, lipids and nucleic acids [20, 21]. These results suggest that denaturation of nucleic acid by the AHP disinfectant occurred to a lesser degree at lower temperatures than at higher temperatures, but this difference did not affect its ability to inactivate PEDV as demonstrated by the swine bioassay results. These results are consistent with those from previous studies where disinfection of contaminated metal surfaces with oxidizing disinfectants inactivated PEDV but did not consistently produce negative PEDV RT-qPCR results after disinfection [22, 23]. Therefore, PEDV-positive RT-qPCR results on environmental samples should be expected when the AHP disinfectant is utilized under freezing conditions, but this does not necessarily indicate that an infectious dose of PEDV remains in the trailer.

Livestock trailers have many non-smooth surfaces and are more complex than the coupons utilized in this study. Trailers contain many channels, grooves, rough surfaces, hinges, latches, and corners that organic material can build up on and provide areas for a virus to be missed by a disinfectant. While, it was understood the smooth aluminum coupons do not replicate all of the surfaces found within livestock trailers, performing a study of this magnitude would not have been feasible with full-size trailers, so the coupons were utilized as a model. The ease with which the 15.24 X 15.24 X 2.54 cm aluminum coupons could be handled made it possible to contaminate the coupons, perform the treatments, collect the inoculum and inoculate pigs for the bioassay for all study groups in less than 1 day. The model also enabled the investigators to stagger the start time for each treatment group so that the pigs could be inoculated immediately after the inoculum was collected, thereby eliminating the need to attempt to neutralize the AHP disinfectant after the 40 min or 60 min contact time.

Livestock trailers are frequently bedded with wood shavings prior to transporting pigs. Incorporation of wood shavings into the model was considered however, the size and type of shavings used for bedding varies widely across the industry and previous work demonstrated that some wood has virucidal properties [24]. Therefore, the aluminum coupons were contaminated with swine feces alone to avoid the possibility of confounding the results with the effect of choice of wood shavings.

While the AHP disinfectant utilized in this study is labeled as virucidal in 5 min with dilutions of 1:16 to 1:64, this study only evaluated two dilutions (1:16 and 1:32) and used considerably longer contact times (40 and 60 min). The longer contact times were chosen because the conditions in this study were less favorable than those used in determining the label and in a previous study which evaluated its efficacy at 20 °C [13]. Further research on the efficacy of an AHP disinfectant under other adverse conditions such as shorter contact times, greater dilution rates, and on perpendicular surfaces to simulate trailer sidewalls is warranted.

## Conclusions

In cold weather months when a complete wash, disinfection, and dry cannot be accomplished, due to lack of resources or other logistical constraints, the results of this study suggest that scraping livestock trailers to remove as much organic material as possible followed by disinfection with at minimum a 1:32 concentration of AHP disinfectant in a solution with 10% PG with at least 40 min of contact time, may be used, as an alternative to doing nothing, to reduce the risk of PEDV transmission associated with livestock trailers.

The results also suggest that a PEDV-positive RT-qPCR result on an environmental sample should be expected after 60 min of contact time when the AHP disinfectant is applied under freezing conditions, but does not necessarily indicate that an infectious dose of PEDV remains in the trailer. Obtaining a negative real-time RT-qPCR result on an environmental sample after disinfection is largely dependent on the type of disinfectant used and the conditions under which it was applied.

## Additional file

Additional file 1: Results of PEDV N-gene RT-qPCR tests on environmental samples from coupons and rectal swabs from pigs used for bioassay. Results from all diagnostic tests conducted for the study are reported for each replicate, one row for each replicate. Test results, Ct value and genomic copies are reported for PCR tests on pre and post treatment environmental samples from coupons. Test result and Ct values are reported for PCR tests on rectal swabs from pigs 3 and 7 days post inoculation for the bioassay. (XLSX 12 kb)

## Abbreviations

AHP: Accelerated hydrogen peroxide
ANOVA: Analyses of variance
ELISA: Enzyme-linked immunosorbent assay
IFA: Indirect fluorescent antibody
ISU VDL: Iowa State University Veterinary Diagnostic Laboratory
N: Nucleocapsid
NPB: National Pork Board
PED: Porcine epidemic diarrhea
PEDV: Porcine epidemic diarrhea virus
PG: Propylene glycol
PRCV: Porcine respiratory corona virus
PRRSV: Porcine reproductive and respiratory syndrome virus
RT-qPCR: Real-time reverse transcription quantitative polymerase chain reaction
TADD: Thermo-assisted drying and decontamination
TGEV: Transmissible gastroenteritis virus
